# Scopolamine Impairs Appetitive But Not Aversive Trace Conditioning: Role of the Medial Prefrontal Cortex

**DOI:** 10.1523/JNEUROSCI.3308-16.2017

**Published:** 2017-06-28

**Authors:** Marie-Astrid Pezze, Hayley J. Marshall, Helen J. Cassaday

**Affiliations:** School of Psychology, University of Nottingham, Nottingham NG7 2RD, United Kingdom

**Keywords:** appetitive, conditioned emotional response, medial prefrontal cortex, rat, scopolamine, trace conditioning

## Abstract

The muscarinic acetylcholine receptor is an important modulator of medial prefrontal cortex (mPFC) functions, such as the working memory required to bridge a trace interval in associative leaning. Aversive and appetitive trace conditioning procedures were used to examine the effects of scopolamine (0.1 and 0.5 mg/kg, i.p.) in male rats. Follow-up experiments tested the effects of microinfusion of 0.15 μg of scopolamine (0.075 μg of in 0.5 μl/side) in infralimbic (IL) versus prelimbic regions of rat mPFC, in appetitive trace and locomotor activity (LMA) procedures. Systemic scopolamine was without effect in an aversive trace conditioning procedure, but impaired appetitive conditioning at a 2 s trace interval. This effect was demonstrated as reduced responding during presentations of the conditioned stimulus (CS) and during the interstimulus interval (ISI). There was no such effect on responding during food (unconditioned stimulus, US) responding or in the intertrial interval (ITI). In contrast, systemic scopolamine dose-relatedly increased LMA. Trace conditioning was similarly impaired at the 2 s trace (shown as reduced responding to the CS and during the ISI, but not during US presentations or in the ITI) after infusion in mPFC, whereas LMA was increased (after infusion in IL only). Therefore, our results point to the importance of cholinergic modulation in mPFC for trace conditioning and show that the observed effects cannot be attributed to reduced activity.

**SIGNIFICANCE STATEMENT** Events are very often separated in time, in which case working memory is necessary to condition their association in “trace conditioning.” The present study used conditioning variants motivated aversively with foot shock and appetitively with food. The drug scopolamine was used to block muscarinic acetylcholine receptors involved in working memory. The results show that reduced cholinergic transmission in medial prefrontal cortex (mPFC) impaired appetitive trace conditioning at a 2 s trace interval. However, scopolamine was without effect in the aversive procedure, revealing the importance of procedural differences to the demonstration of the drug effect. The finding that blockade of muscarinic receptors in mPFC impaired trace conditioning shows that these receptors are critical modulators of short-term working memory.

## Introduction

Trace conditioning procedures examine the ability to associate a conditioned stimulus (CS, e.g., noise) with an unconditioned stimulus (US, e.g., foot shock or food) across an intervening time interval between these events (Pavlov, 1927). This ability relies on an aspect of working memory necessary for the maintenance of information “online” before encoding (Goldman-Rakic and Brown, 1981; Miller et al., 1996; Levy and Goldman-Rakic, 2000; Curtis and D'Esposito, 2003). In delay conditioning, the CS may be of relatively long duration, but there is no time gap between CS offset and US delivery, so the demand on working memory is less. Trace conditioning, but not delay conditioning, is impaired by a number of drug treatments, including the use of the muscarinic acetylcholine (ACh) antagonist scopolamine in eye-blink (Kaneko and Thompson, 1997), fear conditioning (Hunt and Richardson, 2007), and appetitive procedures (Seager et al., 1999).

Medial prefrontal cortex (mPFC) has been identified as a key neural substrate of trace conditioning (Kronforst-Collins and Disterhoft, 1998; Weible et al., 2000, 2003; McLaughlin et al., 2002; Takehara-Nishiuchi et al., 2005) and there is evidence to suggest that the muscarinic ACh receptor is a modulator of this aspect of mPFC function from (850 ms trace) eye blink (Chen et al., 2014) and appetitive (10 s trace) procedures (Flesher et al., 2011).

The ability to associate stimuli over trace intervals and thus link discontiguous events has been argued to reflect temporal information processing, an aspect of relational learning that represents a key component of working memory (Sweatt, 2004). Consistent with the validity of the behavioral model, trace conditioning declines with normal aging in a number of species from rats (McEchron et al., 2004; Moyer and Brown, 2006) to humans (Knuttinen et al., 2001; Cheng et al., 2010). The cholinergic system remains an important focus of aging research (Levey, 1996; Davis et al., 2010) and, although scopolamine has acknowledged limitations as a research tool, it is still widely held to provide a valid pharmacological model of age-related cognitive impairment (Hasselmo and Sarter, 2011).

To our knowledge, there has been no examination of the role of scopolamine in mPFC at longer trace intervals and no systematic comparison of its effects on trace conditioning in aversive and appetitive test variants (Cassaday et al., 2005; Pezze et al., 2015, 2016). Within mPFC, distinct subregions are distinguishable on the basis of their differing patterns of interconnectivity (Fisk and Wyss, 1999; Heidbreder and Groenewegen, 2003; Vertes, 2004). Moreover, there is also electrophysiological evidence for functional differentiation between prelimbic (PL) and infralimbic (IL) neuronal activity measured during trace conditioning at a 20 s trace interval (Gilmartin and McEchron, 2005; Gilmartin et al., 2014).

Experiment 1 of the present study used an established conditioned emotional response (CER) trace procedure (Norman and Cassaday, 2003; Nelson et al., 2012; Pezze et al., 2016) with an extended delay conditioning control group and a relatively short trace interval (Hunt and Richardson, 2007; Baysinger et al., 2012). Specifically, a 10 s CS was presented with or without an equivalent 10 s trace interval as the first step to determine whether any sensitivity to the effects of systemic scopolamine could be demonstrated.

Experiment 2 examined the effects of systemic scopolamine over two trace intervals in an established appetitive procedure in which conditioning has been shown previously to be reduced at a 2 s trace interval after treatment with SKF81297, both systemic and intra-mPFC (Pezze et al., 2015). Experiment 3 tested the effects of the same scopolamine doses on locomotor activity (LMA). Experiment 4 went on to examine the effects of scopolamine administered by microinfusion into PL and IL mPFC subregions at the same coordinates used to examine the role of the dopamine D1 receptor agent SKF81297 (Pezze et al., 2015) and using the trace conditioning variant shown sensitive to the effects of systemic scopolamine in Experiment 2 of the present study.

## Materials and Methods

### 

#### Subjects

Seventy-two experimentally naive male Wistar rats were used in Experiments 1 and 2 (Charles River Laboratories; weights on arrival in the range of 153–206 g and 151–184 g in Experiments 1 and 2, respectively). Allocations to experimental groups were random. One of the rats to be used in Experiment 2 (allocated to the saline 2 s trace group) was humanely killed for reasons unrelated to the experimental procedure. Experiment 3 tested 33 rats (at 320–405 g, counterbalanced for previous experimental group). Throughout the experiments, rats were housed four per cage on a 12:12 h dark/light cycle and received *ad libitum* food and water (up until 24 h before shaping, see below). After arrival, each rat was handled for ∼10 min per day over the course of 1 week. During this time, rats reached mean weight 200 g before the start of any water or food restriction or implantation of cannulae. Experiment 4 used in total 72 rats (operated in the range 277–315 g). Two rats died during surgery due to complications under anesthesia; six were humanely killed because of postoperative complications (two had suspected meningitis; four showed nonspecific signs that might have developed to exceed the moderate severity banding). Synulox was therefore administered subcutaneously (0.05 ml/kg) as a prophylactic measure. Behavioral testing took place after a (minimum) 11 d recovery period. Experiment 5 required 24 of the implanted rats (now at 393–546 g, counterbalanced for previous experimental group).

All procedures were performed in accordance with the principles of laboratory animal care, specifically the UK Animals Scientific Procedures Act of 1986 (Project License PPL 40/3716).

#### Behavioral apparatus

The conditioning boxes have been fully described previously (Norman and Cassaday, 2003; Nelson et al., 2012; Pezze et al., 2016). In the Experiment 1 CER procedure, a waterspout was mounted on 1 wall, 5 cm above the floor and connected to a lickometer supplied by a pump. The CS was a 10 s mixed-frequency (80 dB) noise presented at a 0 or 10 s trace interval. Foot shock of 1 s duration and 1 mA intensity provided the US (pulsed voltage: output square wave 10 ms on, 80 ms off, 370 V peak under no load conditions; MISAC Systems).

In Experiments 2 and 4, otherwise identical conditioning boxes were adapted for appetitive conditioning (for further details, see Pezze et al., 2015). Nose pokes to the food magazine were recorded by the breaking of the photobeam within the food magazine. The US was two 45 mg sucrose pellets dispensed into the magazine (Formula F; Noyes Precision Food). The CS was a 72 dB mixed-frequency noise of 5 s duration. In both aversive and appetitive-conditioning procedures, an experimental background stimulus was provided by 3 wall-mounted stimulus lights and the house light flashing on (0.5 s) and off (0.5 s), continuously for the duration of the conditioning session.

In Experiments 3 and 5, LMA was measured in a dimly lit (50–70 lux) room in 11 clear Perspex chambers (Photobeam Activity System; San Diego Instruments) as described previously (Jones et al., 2011; Pezze et al., 2015). Two consecutive breaks of adjacent beams within the lower level of photobeams generated a locomotor count.

#### Experiments 1–3: systemic injections

**Figure 1.**
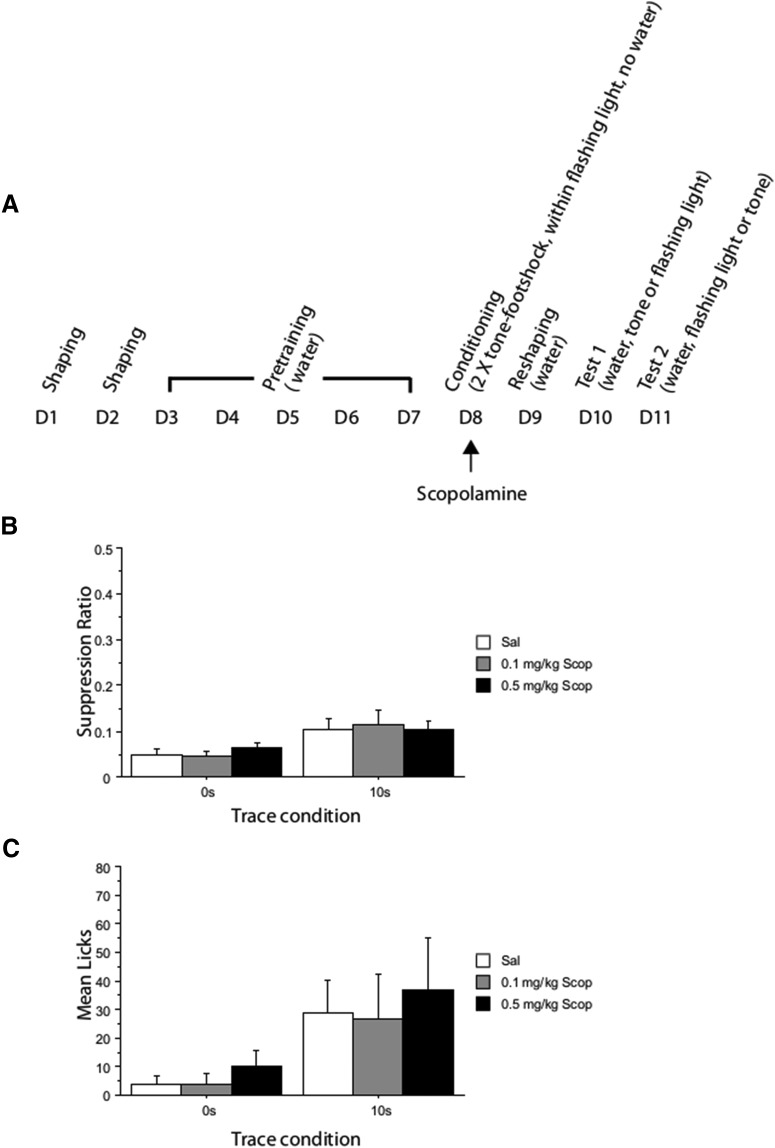
Conditioned suppression to the noise CS in 0 and 10 s conditioned groups after conditioning under saline (Sal) or after systemic (i.p.) treatment with scopolamine at 0.1 mg/kg (Scop 0.1 mg/kg) or 0.5 mg/kg (Scop 0.5 mg/kg) in Experiment 1. Error bars indicate SEM. ***A***, Timeline illustrating the experimental procedures (1 d of conditioning under scopolamine) leading up to the drug-free tests of conditioned suppression. ***B***, Mean suppression ratios. ***C***, Mean number of licks in the first 1 min of the noise test.

**Figure 2.**
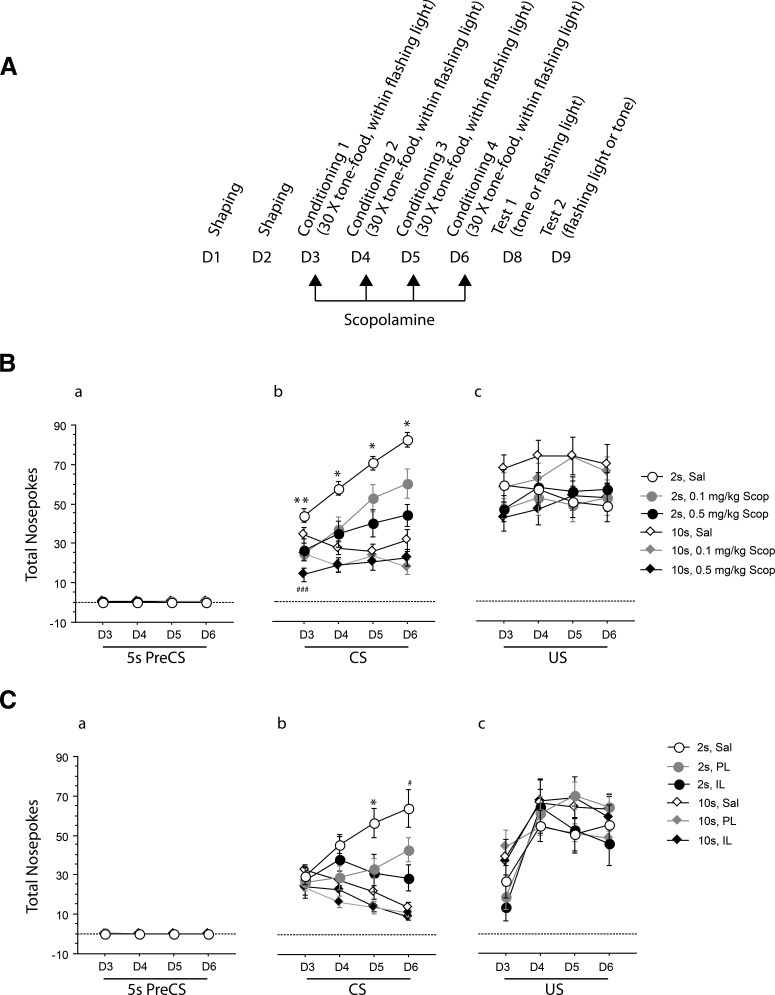
Mean nose pokes are shown as a function of the 4 d of conditioning (D3–D6) for the 5 s of the ITI just before CS presentation (Pre-CS) compared with during the 5 s CS presentation or in the 5 s post-CS when food was delivered (US). Error bars indicate SEM. ***A***, Timeline illustrating the experimental procedures including the 4 d of conditioning under scopolamine. ***B***, Diamonds denote rats conditioned at the 10 s trace interval and circles denote rats conditioned at the 2 s trace interval after systemic treatment with scopolamine (i.p.) at 0.1 mg/kg (gray fill) or 0.5 mg/kg (black fill) in Experiment 2. Control rats (white fill) were injected with saline. Asterisks indicate a significant difference between saline and scopolamine-injected 2 s trace groups with *p < 0.05, **p < 0.01. Hash signs indicate a significant difference between the 0.5 mg/kg-10 s and Sal-10 s groups, ###*p* < 0.001. ***C***, Diamonds denote rats conditioned at the 10 s trace interval and circles denote rats conditioned at the 2 s trace interval after scopolamine infusion (0.075 μg in 0.5 μl/side) in PL (gray fill) or IL (black fill) in Experiment 4. Control rats (white fill) were injected with saline in PL or IL. Asterisk indicates a significant difference between saline and scopolamine-infused 2 s trace groups with *p < 0.05. Hash sign indicates a significant difference between the IL-2 s and Sal-2 s groups, #*p* < 0.05.

Systemic scopolamine doses were based on those found previously to be effective in a trace fear conditioning procedure (Hunt and Richardson, 2007). Scopolamine (Tocris Bioscience) was dissolved in saline (0.9% NaCl) to provide an injection volume of 1 ml/kg. In Experiments 1 and 2, scopolamine (0.1 and 0.5 mg/kg) or saline was injected intraperitoneally 15 min before conditioning sessions. There was one such session in Experiment 1 (Fig. 1*A*). The plasma half-life of scopolamine in the rat is ∼20 min (Lyeth et al., 1992) and the strength of conditioning to the experimental stimuli was tested drug-free on subsequent days of the procedure. There were 4 conditioning sessions and thus 4 d of injections in Experiment 2: conditioning was examined both on the baseline and in follow-up drug-free extinction tests (Fig. 2*A*). In Experiment 3, rats were immediately replaced in the LMA apparatus to document the onset and course of the scopolamine treatments effects.

#### Experiments 4 and 5: microinfusions in the mPFC

Rats were anesthetized using isoflurane delivered in oxygen (induction: 4–5%; maintenance: 1–3%) and secured in a stereotaxic frame. Bilateral infusion guide cannulae (the “mouse” model C235GS-5–1.2; Plastics One) were implanted through small holes drilled in the skull. The tips of the guide cannulae were aimed 0.5 mm above the injection sites in the PL or IL subregion of mPFC. The coordinates for PL were 3 mm anterior and ±0.6 mm lateral from bregma and 4.0 mm ventral from the skull surface; the coordinates for IL were +3 mm anterior and ±0.6 mm lateral from bregma and 5.0 mm ventral from the skull surface (for further details, see Pezze et al., 2015). Cannulae were secured to the skull with dental acrylic and stainless-steel screws. Double stylets (33 gauge; Plastics One) were inserted and closed with a dust cap. During the recovery period, rats were checked daily and habituated to the manual restraint necessary for the scopolamine microinfusions.

The doses of scopolamine used for the microinfusions were based on those found previously to be effective in tests of working memory (Granon et al., 1995; Chudasama et al., 2004; Barker and Warburton, 2009). Rats were gently restrained and 33-gauge injectors (Plastics One) were inserted into the guides. The injector tips extended 0.5 mm below the guides into the PL or IL mPFC and the injector ends were connected through polyethylene tubing to 5 μl syringes mounted on a microinfusion pump (for further details, see Pezze et al., 2015). A volume of 0.5 μl/side of 0.9% saline or of scopolamine in saline was then infused bilaterally over 1 min. The solution used was dissolved at a concentration of 0.15 μg/μl and then aliquoted and kept frozen until use (in the present study, not longer than 3 weeks) at a dose of 0.075 μg of scopolamine per side, in total 0.15 μg per rat. In Experiment 4, the conditioning session started 10 min after the infusion. There were 4 conditioning sessions and thus 4 d of infusions in Experiment 4: conditioning was examined both on the baseline and in follow-up drug-free extinction tests (Fig. 2*A*). In Experiment 5, rats were immediately replaced in the LMA apparatus to document the onset and course of the effects of scopolamine infusion.

#### Experiment 1: CER trace conditioning procedure

Water restriction was introduced 1 d before shaping and there was 1 h *ad libitum* access to water in their home cage after each of the procedural stages outlined below. Rats were trained, conditioned, and tested after 20–23 h of water restriction (fully described previously: Norman and Cassaday, 2003; Nelson et al., 2012; Pezze et al., 2016).

##### Preconditioning to establish baseline lick responses.

Rats were shaped to drink from the waterspout over 2 d. There then followed 5 d of pretraining, in which each rat drank in its allocated conditioning boxes for 15 min each day (timed from first lick). The licking spout was illuminated throughout, but no other stimuli were presented.

##### Conditioning with foot shock.

No water was available within the boxes and the waterspouts were not illuminated. The US foot shock was delivered following termination of the CS in each of two conditioning trials per conditioning session (of which there were two). The first pairing of CS and US was presented after 5 min had elapsed and the second pairing was 5 min after the first, followed by a further 5 min left in the apparatus. In the trace conditioned group, there was a 10 s interstimulus interval (ISI) between the noise (CS) and foot shock (US). In the delay-conditioned control group, there was no interval between the CS and US (0 s ISI). The flashing light experimental background was presented for the duration of the conditioning session within which the two conditioning trials took place. In the absence of licking, there were no behavioral measures to record.

##### Reshaping after foot shock.

On the day after conditioning, animals were reshaped, drug free, and following the same procedure as in the preconditioning sessions. This both reestablished licking after conditioning and provided a measure of contextual conditioning, reflected in the extent to which licking was suppressed in the conditioning boxes.

##### CER tests.

Conditioned suppression to the CS and flashing light background stimulus was tested drug free and in a counterbalanced order. On the first test day, 24 h after reshaping and 48 h after conditioning, the animals were placed in the conditioning boxes and presented with the noise CS or flashing light background stimulus. Water was available throughout the test and the waterspout was illuminated. Once the animals had made 50 licks, the CS was presented for 15 min. On the second test day, 72 h after conditioning, the alternate flashing lights background or noise CS was presented after 50 licks and continued for 15 min. The latency to make 50 licks in the absence of the stimulus (A period) was compared with the time taken to complete 50 licks following stimulus onset (B period) in a suppression ratio: A/(A + B).

#### Experiment 2: appetitive trace conditioning procedure

One day before shaping, food was removed in the morning and the rats were introduced to sucrose pellets in the home cage later the same day before being fed standard laboratory chow rationed at 5 g/100 g body weight. This level of food restriction was continued for the duration of the appetitive conditioning procedure (fully described previously: Pezze et al., 2015).

##### *Pre*conditioning.

Rats were shaped to take reward pellets from the magazine. Then followed 2 d of baseline sessions, during which there were 30 unsignaled rewards (each of 2 sucrose pellets) over 60 min delivered on a variable interval of ∼2 min.

##### Conditioning.

There were 4 d of conditioning at 30 trials per day, delivered within 60 min conditioning sessions. Depending on the experimental group, the reward (US) was delivered 2 or 10 s after CS offset (in the 2 different trace groups). On each conditioning day, 30 signaled rewards were presented on a variable interval. Continuous presentation of the background stimulus (flashing lights) also encompassed the 2 or 10 s ISI. The ISI added to the overall duration so that conditioning sessions lasted 61 or 65 min in total. Extinction tests of conditioning to the CS (noise) and experimental background (flashing lights) stimuli were conducted in a counterbalanced order; 24–48 h after conditioning procedures had been completed. In each case, there were 30 5 s presentations of the stimulus under test over the course of a 60 min session. The number of nose pokes in the different components of the session was recorded.

#### Experiments 3 and 5: locomotor activity procedure

On the day before the LMA tests, each rat was habituated to its allocated activity chamber for 30 min (drug free). On the test day, there was a further 30 min habituation. Each rat was then removed for systemic injection or microinfusion with scopolamine or saline and then replaced in the allocated activity chamber. Total locomotor counts were recorded for each consecutive 10 min epoch for a further 60 min. In Experiment 3, LMA was reassessed in the same way 24 h later over the course of a 30 min test session to determine whether the systemic scopolamine treatments had any residual effects on LMA at this interval.

#### Design and analysis

Experiments 1, 2, and 4 each had 6 experimental groups run in 2 × 3 factorial designs: trace condition (at levels 0 or 10 s in Experiment 1, and 2 or 10 s in Experiments 2 and 4) and drug at levels (saline, 0.1, or 0.5 mg/kg scopolamine in Experiments 1–3; saline, IL, or PL scopolamine in Experiments 4 and 5). In Experiment 1, the dependent variables were drink latencies from the preconditioning day and the number of licks made during the first 1 min of the 15 min session (min-1 licks) to check for differences by experimental condition-to-be, reshaping drink latencies and min-1 licks to assess contextual conditioning to the box cues, suppression ratios and min-1 licks to assess the levels of conditioning to the CS and experimental background stimulus.

In Experiments 2 and 4, the dependent variable was in each case the number of nose pokes into the food magazine and there was an additional repeated-measures factor of days (at four levels). The strength of associative learning was measured as responding during the 5 s presentation of the CS. Significant three-way interactions were followed up by simple main-effects analyses and further *post hoc* tests were by Fisher's LSD test. To compare effects on motivation for food reward or motor responding, similar analyses were conducted on responding during the 5 s in which the US was delivered and on ITI responding in the remainder of the session (excluding the ISI).

In addition, the responding of the animals during the trace interval between CS offset and US delivery was also examined separately for the 2 and 10 s conditioned groups because the intervals in use were not directly comparable. Moreover, the 10 s ISI was broken down into 5 (2 s) bins of time and analyzed using repeated-measures ANOVAs by bins and days. Finally, extinction day test results were examined using the same between-subjects design.

In Experiments 3 and 5, the LMA data were analyzed in blocks of 10 min at 3 levels for the test day habituation, 6 levels under drug, and 3 levels for the test of residual drug action conducted 24 h later on the following day in Experiment 3. *Post hoc* tests were by Fisher's LSD test.

## Results

### Experiment 1: systemic scopolamine in the CER procedure

#### Preconditioning: baseline licking

Min-1 licks were very closely matched across the experimental conditions-to-be (in the range of 252–281 ± 18 licks). Statistically, there were no effects of trace or drug condition-to-be (maximum *F*_(2,66)_ = 2.137, *p* = 0.126 for the interaction on the latency measure).

#### Reshaping: contextual conditioning

There was a main effect of drug on the latency to start drinking at reshape (*F*_(2,66)_ = 3.553, *p* = 0.034). This arose because rats previously conditioned under scopolamine took less time to start drinking at reshape than those previously conditioned under saline. This difference was significant after scopolamine at 0.5 mg/kg (*p* = 0.014) and marginal at 0.1 mg/kg (*p* = 0.050). No other effects were significant (maximum *F*_(1,66)_ = 2.945, *p* = 0.091) for the main effect of trace on the reshape latency.

#### CER tests: CS (noise)

There was a main effect of trace on both the suppression ratio (Fig. 1*B*) and the min-1 licks measure (minimum *F*_(1,66)_ = 7.168, *p* = 0.009; Fig. 1*C*). However, there was no effect of drug (all *F* < 1).

#### CER tests: experimental background (flashing lights)

There was no effect of trace (both *F* < 1) and no effect of drug (maximum *F*_(2,66)_ = 1.020, *p* = 0.366) on the suppression ratio.

### Experiment 2: systemic scopolamine in the appetitive procedure

#### ITI responding

ANOVA of magazine activity between conditioning trials showed no effects of trace or drug (maximum *F*_(2,65)_ = 2.092, *p* = 0.132). Figure 2*Ba* shows the 5 s ITI just preceding the 5 s CS presentations for direct comparison with subsequent CS and US responding. As can be seen in the figure, the response rates in the 5 s ITI immediately pre-CS were very low and unsuitable for ANOVA.

#### CS responding

There were significant main effects of both trace (*F*_(1,65)_ = 63.104, *p* < 0.001) and drug (*F*_(2,65)_ = 13.339, *p* < 0.001). Importantly, the three-way interaction among days, trace, and drug was significant (*F*_(6,195)_ = 3.155, *p* = 0.006) and was also significant in the linear trend (*F*_(2,65)_ = 5.516, *p* = 0.006), so the rate of acquisition depended on the rats' drug group and on the trace interval in use. Figure 2*Bb* shows depressed acquisition in the 2 s trace conditioned groups treated with scopolamine. Follow-up analysis confined to the 2 s trace group confirmed the effect of drug (*F*_(2,32)_ = 9.158, *p* < 0.001). CS responding was reduced under scopolamine compared with saline over the 4 consecutive days of conditioning (day 1: *F*_(2,32)_ = 5.605, *p* = 0.008; saline vs 0.1 mg/kg, *p* = 0.004; saline vs 0.5 mg/kg, *p* = 0.008; day 4: *F*_(2,32)_ = 10.223, *p* < 0.001; saline vs 0.1 mg/kg, *p* = 0.013; saline vs 0.5 mg/kg, *p* < 0.0001). Although analysis confined to the 10 s trace group also showed a significant main effect of drug (*F*_(2,33)_ = 3.969, *p* = 0.028), this effect was restricted to a decrease in the number of nose pokes on day one after injection of 0.5 mg/kg scopolamine (*F*_(2,33)_ = 8.542, *p* = 0.001; saline vs 0.5 mg/kg, *p* < 0.001).

#### US responding

In contrast to the results seen on CS responding, there were no overall effects of trace or drug (maximum *F*_(1,65)_ = 2.107, *p* = 0.151). The US responding showed some fluctuation over the course of acquisition (*F*_(3,195)_ = 3.404, *p* = 0.019). The only significant effect of drug was seen in interaction with days in the linear trend (*F*_(2,65)_ = 3.268, *p* = 0.044) because of increased US responding in scopolamine groups (Fig. 2*Bc*).

#### ISI responding: 2 s trace group

As would be expected, responding in the ISI increased over the course of acquisition, which was confirmed statistically by the main effect of days (*F*_(3,96)_ = 49.279, *p* < 0.001) and in the linear trend (*F*_(1,32)_ = 99.695, *p* < 0.001). Consistent with the effects seen on CS responding, there was both a main effect of drug (*F*_(2,32)_ = 14.181, *p* < 0.001) and an interaction between days and drug (*F*_(6,96)_ = 4.035, *p* = 0.001), which was also significant in the linear trend (*F*_(2,32)_ = 7.010, *p* = 0.003). Treatment with scopolamine depressed ISI responding in a dose-related fashion and the increase in responding over days was less steep (Table 1).

**Table 1.** Mean total nose-poke responding in Experiments 2 and 4

	2 s ISI			10 s ISI		
Experiment 2						
	Saline	Scopolamine, mg/kg, i.p.		Saline	Scopolamine, mg/kg, i.p.	
		0.1	0.5		0.1	0.5
Day 1	8.545 ± 1.442	5.083 ± 1.362	5.167 ± 1.507	15.500 ± 2.445	12.833 ± 1.882	17.667 ± 4.302
Day 2	23.909 ± 1.703	10.667 ± 2.775	7.250 ± 2.669	33.583 ± 8.685	12.333 ± 2.261	11.083 ± 2.675
Day 3	31.182 ± 2.040	19.917 ± 2.832	12.750 ± 3.527	49.000 ± 10.708	16.750 ± 2.273	16.083 ± 4.719
Day 4	32.909 ± 1.975	22.000 ± 2.840	13.167 ± 2.928	56.333 ± 12.154	20.667 ± 5.755	13.917 ± 3.349

Experiment 4						
	Saline	Scopolamine, 0.075 μg in 0.5 μl/side		Saline	Scopolamine, 0.075 μg in 0.5 μl/side	
		IL	PL		IL	PL
Day 1	4.364 ± 1.357	2.625 ± 1.009	3.000 ± 1.141	9.667 ± 2.845	12.600 ± 2.849	13.714 ± 2.653
Day 2	12.545 ± 2.060	5.875 ± 3.204	4.167 ± 1.471	10.667 ± 3.308	8.200 ± 2.235	6.071 ± 1.862
Day 3	21.182 ± 1.672	10.750 ± 3.759	11.167 ± 3.263	18.750 ± 3.810	7.778 ± 2.087	5.714 ± 1.203
Day 4	24.909 ± 3.543	12.875 ± 3.948	18.000 ± 3.382	23.000 ± 6.438	4.667 ± 1.236	2.300 ± 0.651

Data are shown as mean ± SEM.

#### ISI responding: 10 s trace group

The use of a longer trace interval made no difference to the pattern of effects in the ISI; the results were entirely consistent with those seen in the 2 s trace group. There was a main effect of days (*F*_(3,396)_ = 7.958, *p* < 0.001), which was also significant in the linear trend (*F*_(1,33)_ = 12.677, *p* = 0.001). There was again a main effect of drug (*F*_(2,33)_ = 7.729, *p* = 0.002) and an interaction between days and drug (*F*_(6,396)_ = 5.584, *p* < 0.001), which was also significant in the linear trend (*F*_(2,33)_ = 8.505, *p* = 0.001). Treatment with scopolamine overall reduced ISI responding and the increase in responding over days was depressed (Table 1). None of the effects involving bins was significant.

#### Extinction tests

Consistent with the pattern of effects seen in acquisition, analyses of the noise extinction tests revealed a main effect of trace in the 5 s post CS (*F*_(1,65)_ = 4.730, *p* = 0.033) because of generally higher responding in the 2 s conditioned groups. An effect of trace in the ITI (*F*_(1,65)_ = 6.837, *p* = 0.011) arose because of generally higher responding in the 10 s conditioned groups. The effect of previous drug treatment also carried over to extinction (*F*_(2,65)_ = 16.599, *p* < 0.001), seen as overall reduced responding in the 5 s post CS. There was no effect of trace or drug on any measure in the light extinction tests (maximum *F*_(1,65)_ = 3.105, *p* = 0.083, for ITI responding).

### Experiment 3: systemic scopolamine and locomotor activity

**Figure 3.**
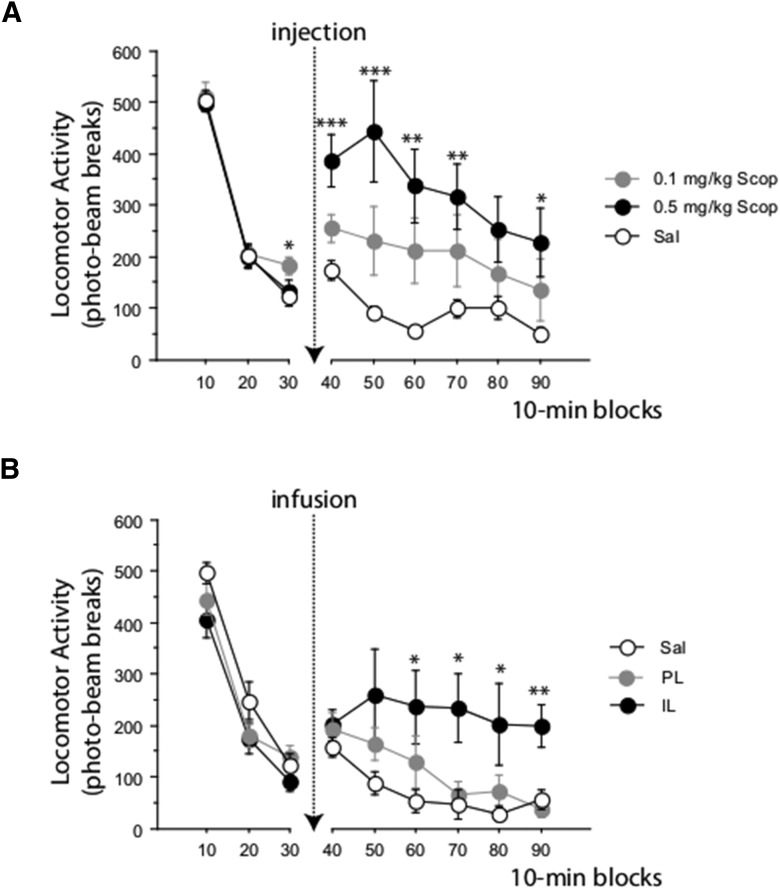
Effect of systemic injections of scopolamine (i.p.) on spontaneous activity. The rats were habituated to the activity chambers for 30 min before any drug treatment. Locomotor activity was then monitored for an additional 90 min. Error bars indicate SEM. Asterisks indicate a significant difference compared with saline with *p < 0.05, **p < 0.01, ***p < 0.0001. ***A***, Spontaneous activity in rats in Experiment 3 after being injected with saline or scopolamine at 0.1 or 0.5 mg/kg (*n* = 11 rats per group). ***B***, Spontaneous activity in rats in Experiment 5 after infusion of saline or scopolamine (0.075 μg in 0.5 μl/side) in PL or IL (*n* = 6–8 rats per group).

LMA levels were well matched before any drug treatment and this was confirmed by analysis of the 30 min habituation session. There was no effect of drug condition-to-be, either overall or in interaction with blocks, both *F* < 1 (Fig. 3*A*). Over the 90 min after drug treatments, there was a clear main effect of drug (*F*_(2,30)_ = 5.531, *p* = 0.009). Scopolamine increased LMA in a dose-related fashion. The difference between rats injected with saline versus 0.1 mg/kg scopolamine was not significant (*p* = 0.112), so the main effect can be attributed to the difference between rats injected with saline versus 0.5 mg/kg scopolamine (*p* = 0.002). Moreover, there was an interaction between drug and blocks (*F*_(16,240)_ = 1.906, *p* = 0.021), which was also significant in the linear trend (*F*_(3,30)_ = 4.740, *p* = 0.016). Figure 3*A* shows that the effect of 0.5 mg/kg peaked 10–20 min postadministration and fell off more sharply thereafter. There was no indication of any residual effect of scopolamine when LMA was reexamined 24 h later (largest *F*_(2,30)_ = 1.640, *p* = 0.211) for the main effect of drug (data not shown).

### Experiment 4: scopolamine infusion in mPFC in the appetitive procedure

#### Histology

**Figure 4.**
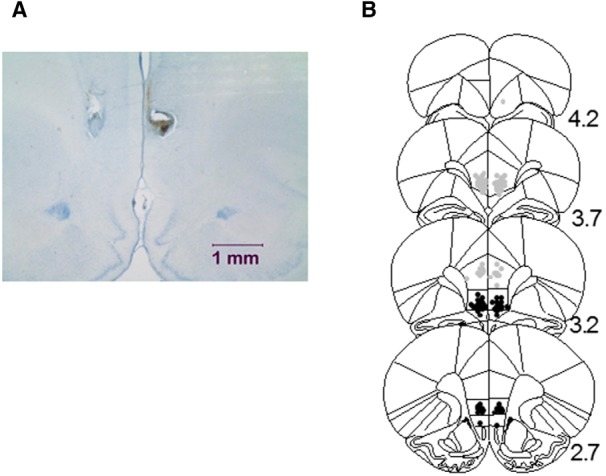
Histological verification of cannula placements for rats used in Experiments 4 and 5. ***A***, Photograph of a representative placement illustrating the area around the injection. There is relatively little indication of gliosis as a result of the microinfusions. ***B***, Approximate locations of infusion cannula tips in the prelimbic (gray dots) and infralimbic (black dots) subregions of the mPFC. Placements are shown on coronal plates adapted from Paxinos and Watson (1998), with numbers indicating distance from bregma in millimeters.

In total, three rats were excluded because the cannula tips were misplaced. Two rats with placements on the borderline of IL were retained in the analyses. This left a final sample size of 61 (*n* = 8–12/cell: 11 Sal-2 s; 11 Sal-10 s; 8 IL-2 s; 9 IL-10 s; 12 PL-2 s; 10 PL-10 s). As shown in Figure 4*A*, there was little evidence of gliosis at the cannula tip. Figure 4*B* illustrates the full range of cannula placements included in the study.

#### ITI responding

As was the case in Experiment 2 (after systemic administration), there was no overall effect of scopolamine microinfusions in IL or PL (*F*_(2,55)_ = 0.067, *p* = 0.936). An interaction between infusion and days (*F*_(6,165)_ = 2.433, *p* = 0.028) was independent of trace. Figure 2*Ca* shows the 5 s ITI just preceding the 5 s CS presentations for direct comparison with subsequent CS and US responding. As can be seen in the figure, the response rates in the 5 s ITI immediately pre-CS were very low and unsuitable for ANOVA.

#### CS responding

The overall effect of scopolamine infusion (*F*_(2,55)_ = 5.444, *p* = 0.007) was to reduce responding during CS presentations. Acquisition was reflected in a days-by-trace interaction (*F*_(3,165)_ = 16.413, *p* < 0.001). Importantly a significant effect of scopolamine infusion was shown in the linear trend of the 3-way interaction (*F*_(2,55)_ = 3.752, *p* = 0.030). Figure 2*Cb* shows that the difference over the 4 d of testing with respect to trace arose because the acquisition functions in the 2 s trace groups infused in IL and PL mPFC subregions were depressed. Follow-up analysis restricted to the 2 s trace group also showed a significant interaction between days and infusion (*F*_(6,84)_ = 2.415, *p* = 0.033). There was no such effect in the 10 s trace group (*F*_(6,81)_ = 0.830, *p* = 0.550). Day-by-day analyses of CS responding confirmed that the effect of infusion developed by day 3 (*F*_(2,28)_ = 4.081, *p* = 0.027) and was also significant on day 4 (*F*_(2,28)_ = 4.949, *p* = 0.014), whereas there was no such an effect of infusion on day 1 (*F*_(2,28)_ = 0.133, *p* = 0.876) or day 2 (*F*_(2,28)_ = 1.319, *p* = 0.283). On day 3, the main effect of infusion was due to a difference between saline and both IL- and PL-infused animals (saline vs IL, *p* = 0.021, saline vs PL, *p* = 0.020). On day 4, the main effect of infusion was due to a difference between the saline and IL groups only (saline vs IL, *p* = 0.040).

#### US responding

In contrast, there was no effect of scopolamine infusion on responding in the 5 s when food was delivered (maximum *F*_(2,55)_ = 2.718, *p* = 0.075; Fig. 2*Cc*).

#### ISI responding: 2 s trace group

There was a main effect of infusion (*F*_(2,28)_ = 4.026, *p* = 0.029). Scopolamine infusion in IL (*p* = 0.019) and PL (*p* = 0.035) reduced responding in the 2 s ISI relative to the level of responding seen in the saline group. As shown in Table 1, this effect was seen regardless of infusion site because the scopolamine groups did not differ (*p* = 0.708).

#### ISI responding: 10 s trace group

The same overall pattern of results was seen in the 10 s trace. There was a main effect of infusion (*F*_(2,27)_ = 4.718, *p* = 0.017), which arose because scopolamine infusion in IL (*p* = 0.046) and PL (*p* = 0.006) overall reduced responding in the ISI relative to the level of responding seen in the saline group, regardless of infusion site because the scopolamine groups did not differ (*p* = 0.447). There was an effect of infusion by days in the 10 s trace group (*F*_(6,81)_ = 4.843, *p* < 0.001), which was also clear in the linear trend (*F*_(2,27)_ = 6.527, *p* = 0.005). Responding within the 10 s trace increased over the 4 d of testing in the saline-infused group, but decreased over the 4 d of testing in the IL and PL infusion groups (Table 1).

However, there was no effect of scopolamine infusion on the distribution of responding within the trace interval in that there were no interactions between infusion and bins (maximum *F*_(24,324)_ = 1.168, *p* = 0.269).

#### Extinction tests

There was a significant interaction between trace and infusion (at the conditioning stage) for both CS (*F*_(2,58)_ = 4.080, *p* = 0.022) and post-CS (*F*_(2,58)_ = 5.898, *p* = 0.005) measures of magazine activity during and after the tone tests. However, there was no effect of prior infusion in the ITI for the tone tests (*F* < 1). Expectation of food was higher in the 2 s than the 10 s trace-conditioned rats treated previously with saline. This difference by trace group was attenuated or even reversed in rats that had been conditioned after infusion of scopolamine in IL or PL. There were no differences by prior infusion on any measures of responding taken during the light extinction tests (maximum *F*_(2,58)_ = 1.011, *p* = 0.370).

### Experiment 5: scopolamine infusion in mPFC and locomotor activity

Scopolamine infusion in mPFC and locomotor activity were tested in a subset of the rats used in Experiment 4, counterbalanced for previous PL–IL infusion condition. One rat could not be tested due to equipment failure and one was subsequently excluded on histological grounds, leaving a final sample size of 22 (*n* = 6–8/cell). There was no effect of infusion condition-to-be on LMA measured during the 30 min test day habituation (*F*_(2,19)_ = 1.823, *p* = 0.189). In contrast to the effects of scopolamine infusion on magazine activity, LMA was increased (*F*_(2,19)_ = 4.673, *p* = 0.022), significantly so in rats receiving the IL infusion (*p* = 0.008) (Fig. 3*B*). The PL and IL infusion groups were also significantly different from each other (*p* = 0.036). The interaction between infusion group and blocks was significant (*F*_(16,152)_ = 1.748, *p* = 0.043).

## Discussion

Experiment 1 used an established CER procedure and a 10 s trace interval suitable to test for conditioning impairment after treatment with the muscarinic ACh antagonist scopolamine (0.1 and 0.5 mg/kg, i.p.). There was some apparent reduction in contextual conditioning measured at the reshape stage of the procedure. However, the results showed no effect of either dose on either 10 s trace conditioning or in the delay conditioning controls. In principle, scopolamine might have altered unconditioned responses to tone or shock and thus obscured effects on trace conditioning. However, any such unconditioned effects would be expected to be equivalent across behavioral conditions and in this sense controlled for. We were interested in differences by trace interval and there was none.

In other aversively motivated procedures, scopolamine has been reported previously to reduce trace conditioning (Kaneko and Thompson, 1997; Hunt and Richardson, 2007) and mPFC has been shown to be an important substrate of ACh modulation of trace conditioning (Flesher et al., 2011; Chen et al., 2014). The present procedure was different from that adopted by Hunt and Richardson (2007) in that we used a fixed rather than adjusted duration CS for the delay conditioning control. However, the CS duration used in the present study was fixed relatively long (at 10 s rather than the 5 s which is typically used in our laboratory for the CER variant) and should have contributed to the working memory load in the trace conditioned group.

In Experiment 2, systemic scopolamine impaired appetitive conditioning at a 2 s trace interval. This effect was demonstrated as reduced responding during presentations of the CS and during the ISI. There was no such effect on responding during food US responding or in the ITI. Moreover, in the Experiment 3 tests of LMA, the same scopolamine treatments dose relatedly increased activity. Experiment 4 and 5 tested the effects of microinfusion of scopolamine (0.075 μg in 0.05 μl/side) in IL versus PL regions of mPFC in the same appetitive trace and LMA procedures. As per the effects of systemic scopolamine, trace conditioning was impaired at the 2 s trace (shown as reduced responding), whereas LMA was increased after infusion in IL mPFC. These results thus point to the importance of cholinergic modulation in mPFC for trace conditioning and show that this effect cannot be attributed to reduced activity.

However, contrary to expectation (Gilmartin and McEchron, 2005; Gilmartin et al., 2014), we did not find evidence for functional differentiation between PL and IL mPFC mediated by action at muscarinic receptors. The same effect of scopolamine was seen after injection at either set of coordinates. The possibility that this lack of difference may relate to diffusion radius is discussed below.

### Selectivity of scopolamine effects

As a muscarinic receptor antagonist, scopolamine is nonselective but nonetheless widely used to assess the role of muscarinic receptors in learning and memory (Klinkenberg and Blokland, 2010; Hasselmo and Sarter, 2011). In the present study, the effects of scopolamine point to the importance of muscarinic receptors in mPFC in appetitive conditioning to a CS presented at a trace interval. However, the behavioral specificity, pharmacological resolution, and neuroanatomical selectivity of the findings should be considered.

Scopolamine effects on delay conditioning were not examined in the appetitive procedure used in Experiments 2 and 4 and cannot be excluded. However, there was no indication that the results were confounded by nonspecific behavioral effects. As shown in Figure 2, direct effects of scopolamine on responding in the ITI and when the US was delivered during the acquisition stage showed a different profile to those seen on CS responding. Moreover, treatment with scopolamine increased LMA in Experiments 3 and 5. Therefore, the reduced CS responding observed in the present study cannot be attributed to generally reduced activity under scopolamine.

In addition to the acknowledged lack of more selective muscarinic antagonists (Klinkenberg and Blokland, 2010), microdialysis studies have shown that the presynaptic actions of muscarinic antagonists can be sufficient to increase the availability of ACh in hippocampus (Dudar, 1977; Nilsson et al., 1990; Newman and Gold, 2016). Consistent with potentially opposing effects on ACh function, LMA can be increased or decreased after treatment with scopolamine (Klinkenberg and Blokland, 2010). Peripheral treatment with scopolamine at 0.15 mg/kg is sufficient to increase hippocampal ACh reliably. However, because this effect peaks only 40 min after injection (Newman and Gold, 2016), it is unlikely to account for the behavioral findings of the present study, which, as shown in Figure 3, were consistent over the 60 min test sessions. With respect to its timeframe of action, Experiment 3 also provided evidence that the behavioral effects of scopolamine were of <24 h duration (because there was no residual effect of prior scopolamine treatment on the following day). In Experiment 2, impaired expression of trace conditioning was confirmed in extinction tests conducted 24 h later, at which point direct effects of scopolamine on activity can be excluded. We cannot exclude the possibility of some confounded residual effect on activity in Experiment 4. The brain elimination half-life may be longer (Ali-Melkkilä et al., 1993) and, in the present study, we observed longer-lasting effects of scopolamine on LMA after infusion into IL (compared with the LMA seen after systemic injection at 0.1 mg/kg). Although the effect of systemic scopolamine on activity dropped off over the 90 min LMA test session, the hyperactivity after infusion in IL was maintained.

With respect to the neuroanatomical selectivity of the microinfusion procedure, the expected diffusion radius of a 0.5 μl injection is ∼1 mm (Allen et al., 2008). However, the minimally pharmacologically active concentrations (at the outer limits of the diffusion radius) are unknown, so the best criterion to determine spread has to be functional (Edeline et al., 2002). In the present study, the fact that there was some difference in the effects of scopolamine in IL versus PL on LMA (in that there was no significant effect of scopolamine on LMA at the coordinates used to target PL) suggests that functional differentiation of IL and PL was achieved with the doses in use.

### Implications of the role of mPFC muscarinic receptors in trace conditioning

The results of the present study suggest that muscarinic receptors within the PL–IL mPFC are important for trace conditioning. This finding is broadly consistent with a variety of other studies showing the effects of scopolamine (Wirsching et al., 1984; Klinkenberg and Blokland, 2010), specifically scopolamine in PFC on aspects of working memory (Granon et al., 1995; Chudasama et al., 2004; Barker and Warburton, 2009; Hasselmo and Sarter, 2011; Major et al., 2015). As a simple associative measure of working memory, trace conditioning is an established model of hippocampal-dependent memory function (Sweatt, 2004). The model has good validity in that the deleterious effects of neurodevelopmental insult can be mitigated by nutritional supplementation with choline to improve cholinergic neurotransmission (Thomas and Tran, 2012). Trace conditioning has also been suggested to provide a behavioral model of age-related decline (Knuttinen et al., 2001; McEchron et al., 2004; Moyer and Brown, 2006; Cheng et al., 2010). Therefore, the further delineation of its neuropharmacological substrates may suggest new targets to counteract the memory loss of aging (Levey, 1996; Davis et al., 2010; Hasselmo and Sarter, 2011).

The finding that the effects of systemic scopolamine were reproduced by microinfusion in mPFC is consistent with the earlier identified role of mPFC in trace conditioning (Kronforst-Collins and Disterhoft, 1998; Weible et al., 2000, 2003; McLaughlin et al., 2002; Takehara-Nishiuchi et al., 2005) and further delineates the neuromodulatory role of ACh. With respect to the wider literature, our findings are also consistent with evidence pointing to the role of ACh in the attentional aspects of short-term memory (Hasselmo and Sarter, 2011; Klinkenberg et al., 2011) and conventional tests of working memory that require flexible choice responding (Graef et al., 2011).

Importantly, we also present evidence that the demonstration of neuromodulatory effects may depend on the trace interval and/or procedural variant in use. Differences due to the trace interval or ISI are in practice difficult to distinguish from other procedural differences such as the number of learning trials and the ITI for tasks, which are motivated appetitively rather than aversively (Pezze et al., 2016). Regardless of the underlying mechanism, we have found evidence that procedural differences set boundary conditions for the demonstration of scopolamine effects on trace conditioning.

The role of mPFC is consistent with other evidence pointing to the importance of muscarinic ACh regulation of aspects of mPFC function (Santini et al., 2012), including working memory (Granon et al., 1995; Chudasama et al., 2004; Barker and Warburton, 2009; Major et al., 2015). However, we submit that this role is likely supported by a wider network of distributed mechanisms (Sarter et al., 2005; Egorov et al., 2006; Esclassan et al., 2009; Hasselmo and Sarter, 2011; Baysinger et al., 2012), including cholinergic projections to perirhinal cortex (Bang and Brown, 2009) and the septohippocampal system (Givens and Olton, 1995; Seeger at al., 2004; Raybuck and Gould, 2010; Newman and Gold, 2016).
